# Age-related changes in monocytes exacerbate neointimal hyperplasia after vascular injury

**DOI:** 10.18632/oncotarget.3881

**Published:** 2015-04-19

**Authors:** Laisel Martinez, Camilo Gomez, Roberto I. Vazquez-Padron

**Affiliations:** ^1^ Department of Surgery and Vascular Biology Institute, University of Miami Miller School of Medicine, Miami, FL, USA

**Keywords:** age, monocytes, gene expression, balloon injury, neointimal hyperplasia

## Abstract

Neointimal hyperplasia is the leading cause of restenosis after endovascular interventions. It is characterized by the accumulation of myofibroblast-like cells and extracellular matrix in the innermost layer of the wall and is exacerbated by inflammation. Monocytes from either young or aged rats were applied perivascularly to injured vascular walls of young recipient animals. Monocytes from aged rats, but not young donors, increased neointima thickness. Accordingly, the gene expression profiles of CD11b^+^ monocytes from aged rats showed significant up-regulation of genes involved in cellular adhesion, lipid degradation, cytotoxicity, differentiation, and inflammation. These included cadherin 13 (*Cdh13*), colony stimulating factor 1 (*Csf1*), chemokine C-X-C motif ligand 1 (*Cxcl1*), endothelial cell-selective adhesion molecule (*Esam*), and interferon gamma (*Ifng*). In conclusion, our results suggest that the increased inflammatory and adhesive profile of monocytes contributes to pathological wall remodeling in aged-related vascular diseases.

## INTRODUCTION

Activation of monocytes and differentiation into macrophages are major steps in vascular proliferative diseases like atherosclerosis and restenosis [1-3]. Monocytes/macrophages produce growth factors that stimulate vascular smooth muscle cell (VSMC) differentiation and growth [4, 5], while contributing to the deposition of extracellular matrix (ECM) to facilitate immune cell infiltration [6, 7] and myofibroblastic migration from the adventitia [8]. It is believed that aged-related changes in monocyte numbers and phenotypes contribute to the development of vascular diseases in the elderly [9-12].

Most recently, we and others demonstrated that aging exacerbates neointimal hyperplasia (NIH) after vascular injury, likely as a consequence of increased infiltration of monocytes [6, 13] and higher sensitivity of VSMC to proliferation stimuli [8]. Interestingly, we also found that monocytes/macrophages in aged rats are more pro-inflammatory and adhesive than in younger animals, which in part explains the increased number of macrophages after vascular injury in aged vasculature [6]. Not surprisingly, pharmacological depletion of monocytes compromises neointima development after arterial injury [6].

This study aims at demonstrating the restenotic properties of aged monocytes in the rat model of vascular injury. We controled for additional age-related interacting factors and demonstrate for the first time that changes in monocytes from aged rats are sufficient to exacerbate NIH after arterial injury. Gene expression profiles of monocytes from young and aged rats revealed significant differences in the expression of adhesion molecules, cytotoxic factors, as well as genes associated with inflammation, cellular differentiation and migration. Altogether, our work identifies potential molecular players that may be involved in the differential contribution of monocytes to post-injury restenosis with aging.

## RESULTS

### Aged rats have higher counts of blood monocytes than younger animals

**Figure 1:**
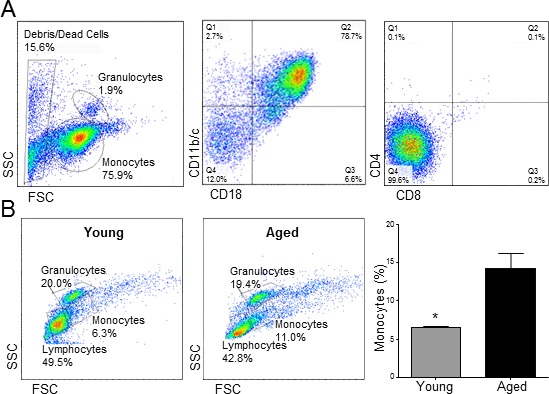
Aging causes monocytosis in rats **A.** Representative flow cytometry analysis of purified monocytes. **B.**Cell counts ± SEM of monocytes, granulocytes and lymphocytes in young and aged rats (*n* = 3 per group). Numbers are expressed as percentages of peripheral blood mononuclear cells. *p < 0.05.

In order to investigate possible age-related changes in monocytes that may explain a differential contribution to NIH after vascular injury, monocytic cells from Fischer rats aged 2 and 22 months old (*n* = 3 per age group) were isolated and purified by negative immunomagnetic separation. Purified live cells were 90% monocytes, of which over 75% stained positive for both CD11b/c and CD18 (Figure 1A). The isolated monocytes were essentially negative for the CD4 and CD8 markers (Figure 1A). The total percentage of monocytes in peripheral blood mononuclear cells (PBMC) was significantly higher in aged rats compared to young animals (14.4 ± 2.0 *vs*. 6.6 ± 0.2%, *p* = 0.02; Figure 1B), as previously reported [13], while the opposite was observed for peripheral lymphocytes, although not statistically significant (43.1 ± 12.3 *vs*. 49.4 ± 21.2%, *p* = 0.8).

### Monocytes from aged rats exacerbate NIH in injured arteries of young animals

Age-related phenotypic differences in human monocytes have been associated with an increased risk for atherosclerosis and restenosis [12, 14, 15]. We investigated whether, in the absence of confounding age-related factors, monocytes from aged rats were sufficient to exacerbate NIH in injured arteries of young animals. To this end, approximately 2×10^6^ monocytes isolated from young and aged animals were delivered perivascularly around injured arteries of young recipients (*n* = 6 per age group). A control group received an equivalent volume of vehicle under the same conditions (*n* = 6).

**Figure 2:**
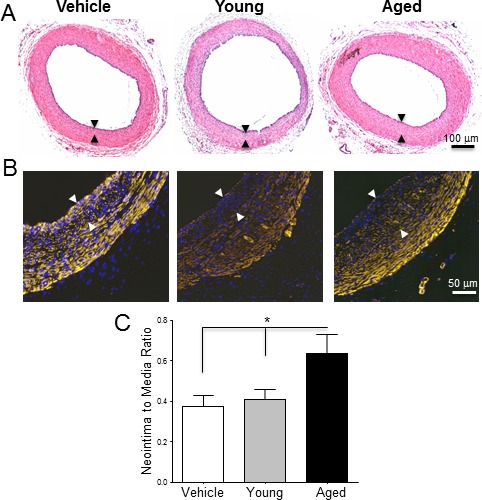
Monocytes from aged rats exacerbate post-injury neointimal hyperplasia in young arteries **A**-**B.** Equal numbers of monocytes from young and aged rats were suspended in Matrigel and exogenously seeded around arteries prior to balloon injury. **A**. Hematoxylin and Eosin and **B.** SMA stained cross-sections of injured arteries treated with Matrigel alone, or monocytes from young and aged donors, and harvested 21 days after injury. The neointima layer is delineated by arrows. **C.** Neointima-to-media ratios of the three experimental groups expressed as mean ratio ± SEM (*n* = 6 per group). *p < 0.05.

**Figure 3:**
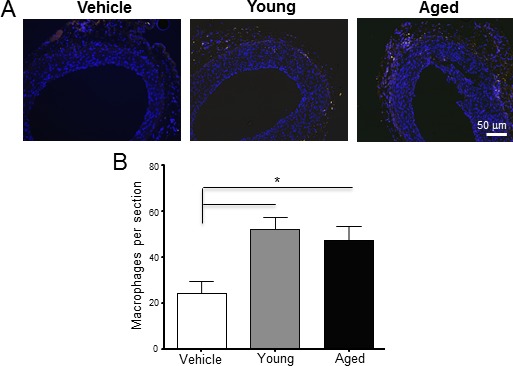
Perivascular delivery of monocytes from young and aged rats increased the number of CD68+ macrophages in the adventitia **A.** Immunofluorescent staining of injured arteries 21 days after surgery using an anti-CD68 antibody. CD68^+^ macrophages are shown in yellow, while nuclei were counter-stained with DAPI (blue). **B.** Number of macrophages per section in the three experimental groups. Bars represent the mean ± SEM (*n* = 4 per group). *p < 0.05.

Arteries that received monocytes from aged animals developed thicker neointimas than those that received cells from young rats (N/M ratio: 0.63 ± 0.09 *vs*. 0.40 ± 0.05, *p* = 0.049; Figure 2). In contrast, monocytes from young animals did not modify neointima thickness with respect to rats receiving vehicle alone (N/M ratio: 0.37 ± 0.05, *p* = 0.7). The number of CD68^+^ macrophages increased significantly in the adventitia of arteries that received monocytes compared to the vehicle control (24.50 ± 5.07 in control *vs*. 47.25 ± 6.29 cells per section in aged, *p* = 0.03; Figure 3A-3B). However, despite the differences observed in neointima thickness measurements between arteries that received cells from young or aged donors, no significant differences in the number of infiltrated macrophages were detected between both experimental conditions (52.00 ± 5.20 *vs*. 47.25 ± 6.29 cells per section, *p* = 0.6; Figure 3B).

### Adhesion molecules and pro-inflammatory factors are differentially expressed in monocytes from aged and young animals

We then looked for differentially expressed genes that could account for the increased restenotic effect of monocytes from aged animals. Table 1 lists significantly up-regulated genes in monocytes from aged rats compared to those from young donors (*n* = 3 per group). Interestingly, this list includes a number of transcripts involved in differentiation (*Csf1, Egr1, Gfi1, Gprc5a, Jun, Rasl11b*), cell survival (*Dapk2, Nol3*), migration (*Lpar3*), cell adhesion (*Asam, Cd24, Cdh13, Chn2, Cldn23, Esam, Mcam, Mpp7*), cytotoxicity (*Gzmbl3, Gzmf*), ECM remodeling (*Cela1*), and inflammation (*Cxcl1, Dusp2, Ifng, Klrb1, Lyzl4, Nr4a2, Nr4a3, Plk2, Plk3, Rgs16*). Numerous genes coding for cytoskeletal proteins (*Actn2, Anxa8, Calb2, Dnah8, Gels, Mark1, Myh2, Scin, Tnni1*), cell cycle modulators (*Btg3, Cdkn2a, Cenpt, Cks1b, Cks2, Gmnn, Kif20b, Plk3, Prc1, Spag5, Spc25*), as well as lipid degradation enzymes (*Pla2g2f, Pnliprp2*) were also up-regulated.

**Table 1:** Significantly up-regulated genes in monocytes from aged vs. young rats

Gene	Description	Fold change	p	FDR
Pnliprp2	Pancreatic lipase-related protein 2	372.49	0.00E+00	1.00E-05
Ispd	Isoprenoid synthase domain-containing protein	366.83	0.00E+00	1.13E-03
Anxa8	Annexin A8	210.29	0.00E+00	1.70E-04
Gzmf	Granzyme F	208.57	0.00E+00	3.24E-03
Sfrp1	Secreted frizzled-related protein 1	195.25	1.00E-05	3.24E-03
Cdh13	Cadherin 13	61.88	0.00E+00	3.10E-04
Lama3	Laminin alpha 3	57.29	3.00E-05	5.99E-03
Calb2	Calbindin 2	52.87	5.00E-05	7.68E-03
Thumpd1	THUMP domain-containing protein 1	40.75	0.00E+00	1.00E-05
Klrb1	Killer cell lectin-like receptor subfamily B member 1	40.66	3.00E-05	6.22E-03
Ppic	Peptidyl-prolyl cis-trans isomerase C	34.04	1.80E-04	1.73E-02
Asam	Adipocyte adhesion molecule	24.46	1.00E-05	3.24E-03
Lyzl4	Lysozyme A	22.60	9.00E-05	1.10E-02
Cdkn2a	Cyclin-dependent kinase inhibitor 2A	21.33	2.00E-05	5.55E-03
Cela1	Chymotrypsin-like elastase family member 1	15.50	6.00E-05	8.58E-03
Lrrc72	Leucine-rich repeat-containing protein 72	12.78	1.00E-05	3.24E-03
Tnni1	Troponin I, slow skeletal muscle	12.60	4.80E-04	3.50E-02
Resp18	Regulated endocrine-specific protein 18	12.48	3.00E-04	2.63E-02
Nr4a3	Nuclear receptor subfamily 4 group A member 3	12.13	1.00E-05	4.56E-03
Dnah8	Dynein, axonemal heavy chain 8	11.74	1.30E-04	1.45E-02
Ephb3	Ephrin type-B receptor 3	11.41	2.00E-05	5.55E-03
Atp1a2	Sodium/potassium-transporting ATPase subunit alpha-2	10.16	2.00E-05	5.55E-03
Mcam	Cell surface glycoprotein MUC18	9.85	2.00E-05	5.35E-03
Mpp7	MAGUK p55 subfamily member 7	8.67	3.00E-05	6.33E-03
Actn2	Actinin alpha 2	8.53	7.80E-04	4.89E-02
Slc4a4	Electrogenic sodium bicarbonate cotransporter 1	8.42	1.00E-05	3.24E-03
Dusp2	Dual specificity protein phosphatase 2	8.19	3.00E-05	6.33E-03
Ifng	Interferon gamma	6.45	2.00E-05	5.43E-03
Spag5	Sperm associated antigen 5	5.99	7.00E-04	4.52E-02
Pask	PAS domain-containing serine/threonine protein kinase	5.93	7.00E-04	4.87E-02
Cldn23	Claudin 23	5.86	7.00E-05	9.12E-03
Gstt3	Glutathione S-transferase theta 3	5.75	5.40E-04	3.79E-02
Gfi1	Zinc finger protein Gfi-1	5.73	4.00E-05	6.77E-03
Lpar3	Lysophosphatidic acid receptor 3	5.65	1.00E-04	1.18E-02
Nol3	Nucleolar protein 3	5.62	1.30E-04	1.43E-02
Gzmbl3	Granzyme B-like 3	5.56	0.00E+00	2.46E-03
Copz2	Coatomer protein complex, subunit zeta 2	5.14	3.20E-04	2.68E-02
Kif20b	M-phase phosphoprotein 1	5.04	6.20E-04	4.15E-02
Nr4a2	Nuclear receptor subfamily 4 group A member 2	5.00	5.00E-05	7.84E-03
Myh2	Myosin, heavy chain 2	4.95	4.50E-04	3.35E-02
Cks1b	Cyclin-dependent kinases regulatory subunit 1	4.83	4.10E-04	3.16E-02
Spc25	Kinetochore protein Spc25	4.72	6.30E-04	4.22E-02
Arhgef39	Rho guanine nucleotide exchange factor 39	4.51	4.00E-04	3.13E-02
Gfra2	GDNF family receptor alpha-2	4.47	5.20E-04	3.71E-02
Plk3	Serine/threonine-protein kinase PLK3	4.47	2.00E-05	5.36E-03
Plk2	Serine/threonine-protein kinase PLK2	4.43	2.00E-05	5.35E-03
Dapk2	Death-associated kinase 2	4.35	3.40E-04	2.78E-02
Agpat3	1-acyl-sn-glycerol-3-phosphate acyltransferase gamma	4.34	2.20E-04	2.00E-02
Tmem246	Transmembrane protein 246	4.33	1.00E-05	4.56E-03
Scin	Adseverin	4.32	1.70E-04	1.69E-02
Esam	Endothelial cell-selective adhesion molecule	4.21	3.60E-04	2.93E-02
Cks2	Cyclin-dependent kinases regulatory subunit 2	4.18	8.00E-05	1.07E-02
Sgca	Alpha sarcoglycan	4.18	3.10E-04	2.66E-02
Rasl11b	Ras-like protein family member 11B	4.15	7.00E-05	9.12E-03
Csf1	Macrophage colony-stimulating factor 1	4.11	4.50E-04	3.35E-02
Fv1	Friend virus susceptibility protein 1	4.07	4.70E-04	3.46E-02
Chn2	Beta-chimaerin	4.01	4.40E-04	3.34E-02
Ube2t	Ubiquitin-conjugating enzyme E2T	3.95	5.10E-04	3.76E-02
Xxylt1	Xyloside xylosyltransferase 1	3.95	6.00E-05	8.91E-03
Srpk3	Serine/arginine-rich splicing factor protein kinase 3	3.90	4.00E-05	6.56E-03
Prc1	Protein regulator of cytokinesis 1	3.82	7.40E-04	4.72E-02
Cxcl1	Chemokine (C-X-C motif) ligand 1	3.76	7.60E-04	4.81E-02
Mfsd4	Major facilitator superfamily domain-containing protein 4	3.74	7.00E-05	9.23E-03
Cd24	Signal transducer CD24	3.68	5.80E-04	3.98E-02
Gprc5a	Retinoic acid-induced protein 3	3.63	3.40E-04	2.80E-02
Mark1	Serine/threonine-protein kinase MARK1	3.61	1.00E-04	1.22E-02
Rgs16	Regulator of G-protein signaling 16	3.61	1.00E-04	1.18E-02
Gmnn	Geminin	3.54	2.00E-04	1.90E-02
Chrne	Acetylcholine receptor subunit epsilon	3.45	1.90E-04	1.80E-02
Calcr	Calcitonin receptor	3.41	7.30E-04	4.66E-02
Sccpdh	Saccharopine dehydrogenase-like oxidoreductase	3.37	4.00E-04	3.14E-02
Pik3r1	Phosphoinositide-3-kinase, regulatory subunit 1 alpha	3.36	5.30E-04	3.77E-02
Ly49i4	Ly49 inhibitory receptor 4	3.35	4.50E-04	3.35E-02
Slco4a1	Solute carrier organic anion transporter family member 4A1	3.23	1.30E-04	1.43E-02
Ppp1r36	Protein phosphatase 1, regulatory subunit 36	3.04	7.00E-04	4.52E-02
Pla2g2f	Group IIF secretory phospholipase A2	3.03	3.20E-04	2.70E-02
Syne1	Spectrin repeat-containing, nuclear envelope protein 1	2.97	6.60E-04	4.34E-02
Egr1	Early growth response protein 1	2.95	7.00E-04	4.52E-02
Gels	Gelsolin	2.92	1.30E-04	1.43E-02
Wdr25	WD repeat-containing protein 25	2.90	1.80E-04	1.73E-02
Prdm1	PR domain zinc finger protein 1	2.82	4.80E-04	3.50E-02
Jun	Transcription factor AP-1	2.73	1.40E-04	1.51E-02
Grk4	G protein-coupled receptor kinase 4	2.42	6.20E-04	4.15E-02
Lmnb1	Lamin B1	2.39	6.20E-04	4.15E-02
Btg3	Protein BTG3	2.19	7.30E-04	4.66E-02

On the other hand, the group of down-regulated genes (or up-regulated in monocytes from young rats compared to aged animals) included transcripts involved in immune regulation (*Tnfrsf17*), chemotaxis (*CCr10, S1pr1*), TLR signaling (*Ticam1*), and antigen presentation (*RT1*; Table 2). Numerous B cell and red blood cell (RBC) related transcripts are also up-regulated in monocytes from young rats. While the latter may be suggestive of contamination with other cell types, other studies have reported similar findings in the past, including the expression of IgM proteins [16].

**Table 2:** Significantly down-regulated genes in monocytes from aged vs. young rats

Gene	Description	Fold change	p	FDR
S1pr1	Sphingosine 1-phosphate receptor 1	0.03	3.20E-04	2.68E-02
Hbe1	Hemoglobin, epsilon 1	0.04	7.40E-04	4.72E-02
Slc4a1	Solute carrier organic anion transporter family member 4A1	0.05	1.70E-04	1.69E-02
Eraf	Alpha hemoglobin stabilizing protein	0.08	5.50E-04	3.83E-02
IGKV	Immunoglobulin kappa light chain V region	0.08	4.00E-05	6.79E-03
IGG2A	Immunoglobulin gamma-2A chain C region	0.10	1.20E-04	1.38E-02
Ccr10	C-C chemokine receptor type 10	0.11	3.00E-05	6.05E-03
Zcchc14	Zinc finger, CCHC domain-containing protein 14	0.12	4.00E-05	6.53E-03
Mzb1	Marginal zone B and B1 cell-specific protein	0.14	2.00E-05	5.35E-03
Tnfrsf17	Tumor necrosis factor receptor superfamily member 17	0.14	1.00E-05	3.24E-03
IGLC2	Immunoglobulin lambda-2 chain C region	0.16	3.00E-05	6.19E-03
Cd79b	B-cell antigen receptor complex-associated protein, beta chain	0.17	1.00E-05	4.64E-03
Pxdc1	PX domain-containing protein 1	0.17	5.00E-05	7.84E-03
Vpreb3	Pre-B lymphocyte protein 3	0.17	3.00E-05	5.99E-03
Olr686	Olfactory receptor Olr686	0.19	1.30E-04	1.43E-02
Itih4	Inter-alpha-trypsin inhibitor heavy chain family, member 4	0.20	8.00E-05	1.01E-02
Slc22a23	Solute carrier family 22 member 23	0.20	2.00E-05	5.55E-03
Derl3	Derlin-3	0.23	1.00E-05	4.56E-03
Rhbdl1	Rhomboid, veinlet-like protein 1	0.23	3.00E-05	6.33E-03
Ms4a1	Membrane-spanning 4-domains, subfamily A, member 1	0.23	1.40E-04	1.46E-02
Cd79a	B-cell antigen receptor complex-associated protein, alpha chain	0.23	1.00E-04	1.20E-02
Eaf2	ELL-associated factor 2	0.24	1.80E-04	1.75E-02
Sspn	Sarcospan	0.24	4.90E-04	3.55E-02
Pde6h	Cone-like cGMP-phosphodiesterase 6 gamma subunit	0.24	2.90E-04	2.58E-02
Try10	Trypsin 10 precursor	0.25	2.10E-04	1.92E-02
Fcer2	IgE Fc receptor, low affinity II, alpha polypeptide isoform b	0.26	1.70E-04	1.72E-02
RT1-CE10	RT1 class I, locus CE10	0.28	8.00E-05	1.07E-02
Myf6	Myogenic factor 6	0.30	1.70E-04	1.72E-02
Gas6	Growth arrest-specific protein 6	0.30	1.40E-04	1.47E-02
Jakmip1	Janus kinase and microtubule-interacting protein 1	0.31	9.00E-05	1.13E-02
Pon1	Serum paraoxonase/arylesterase 1	0.32	6.90E-04	4.50E-02
Dgkg	Diacylglycerol kinase, theta	0.32	4.50E-04	3.35E-02
Txndc5	Thioredoxin domain-containing protein 5, endoplasmic reticulum	0.33	4.00E-05	6.49E-03
RT1-CE3	RT1 class I, locus CE3	0.33	1.80E-04	1.75E-02
RT1-A2	RT1 class I, locus A2	0.33	3.90E-04	3.09E-02
Idh1	Isocitrate dehydrogenase [NADP], cytoplasmic	0.35	5.80E-04	3.98E-02
Ticam1	Toll-like receptor adaptor molecule 1	0.36	3.90E-04	3.09E-02
Hvcn1	Hydrogen voltage-gated channel 1	0.37	3.60E-04	2.95E-02
Sec11c	Signal peptidase complex catalytic subunit SEC11C	0.37	6.00E-05	8.26E-03
Musk	Muscle, skeletal, receptor tyrosine protein kinase	0.38	6.40E-04	4.26E-02
Dgka	Diacylglycerol kinase, alpha	0.39	5.90E-04	4.02E-02
Nol4l	Nucleolar protein 4-like	0.42	6.80E-04	4.45E-02
Mkrn1	Makorin ring finger protein 1	0.43	4.10E-04	3.20E-02
Pdia4	Protein disulfide isomerase-associated 4	0.43	4.30E-04	3.31E-02

**Figure 4:**
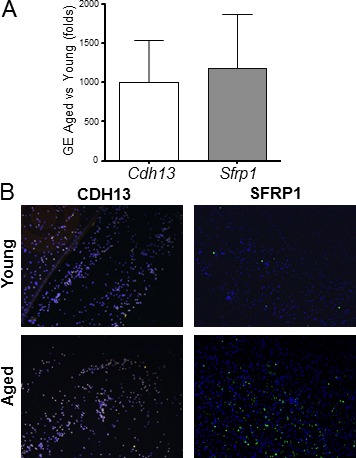
Aging induces significant gene expression differences in rat monocytes **A.** Confirmatory qRT-PCR for cadherin 13 (*Cdh13)* and secreted frizzled-related protein 1 (*Sfrp1)* in monocytes. Gene expression (GE) is expressed in fold change of aged *vs*. young as calculated using the ddCT method. **B.** Immunofluorescent staining of young and aged monocytes using antibodies against CDH13 and SFRP1.

The mRNA and protein expressions of two of the most up-regulated transcripts (*Cdh13* and *Sfrp1*) were confirmed by qRT-PCR and immunofluorescence (Figure 4). Both analyses demonstrate higher expressions of both genes in monocytes from aged rats compared to young animals. Nevertheless, the immunofluorescence staining clearly shows that monocytes isolated from animals of both age groups are heterogeneous in nature, since not all cells stained uniformly for SFRP1 and different levels of fluorescence are evident for CADH13 (Figure 4B).

## DISCUSSION

Numerous studies indicate that aging favors pro-inflammatory phenotypes on monocytes, which predispose for cardiovascular disease and chronic subclinical inflammation [9-12]. In this work we demonstrated that 1) aging causes monocytosis in rats, 2) monocytes from aged animals exacerbate NIH, and 3) aging induces profound differences in monocyte gene expression.

Our results underscore the pro-inflammatory potential of monocytes from aged animals in the vascular response to balloon injury. Perivascular delivery of equal numbers of monocytes from either young or aged donors to young injured arteries showed that cells from aged rats have restenotic properties, in contrast to those from young animals. In addition, the different effects of monocytes from both age groups on neointima development occurred in the absence of other age-related aggravating factors, including monocytosis. In agreement with previous reports [13], we confirmed that a higher percentage of monocytes in PBMC is associated with aging in rats. But more importantly, we showed that, although higher cell counts may contribute to NIH, it is the phenotypic changes in monocytes from aged rats that are truly responsible for inducing constrictive vascular remodeling. The number of infiltrated macrophages in injured arteries was the same at 21 days with monocytes from both groups. This suggests that the different effects on neointima formation were mostly caused by secretable factors and/or that detectable differences in macrophage infiltration occurred at an earlier time point after the procedure. It is also likely that other age-related factors in the vascular wall further stimulate infiltration, differentiation and macrophage survival in injured arteries, as previously demonstrated by our group [6].

The gene expression profile of monocytes from aged rats reveals possible mediators of their restenotic and pro-inflammatory properties. In addition to the numerous up-regulated adhesion molecules (Table 1 and Results), monocytes from aged animals have higher expression of *Csf1, Cxcl1,* and *Ifng*. This combination of cytokines is highly synergistic. As the primary growth factor for the differentiation of monocytes into macrophages, CSF1 is more potent in combination with IFNγ [17]. The latter is a major inflammatory cytokine and contributes to the development of NIH after balloon angioplasty by inducing VSMC proliferation [5]. CXCL1, in turn, amplifies the inflammatory response and stimulates the recruitment of additional monocytes [7]. As an antagonist of the Wnt/β-catenin pathway, SFRP1 is expected to reduce chemotaxis and decrease VSMC proliferation [18]. However, overexpression of this protein is also known to improve cell-to-cell contacts with endothelial cells (EC) and VSMC [19], and it may influence ECM remodeling by up-regulating metalloproteinases [20]. Other up-regulated genes such as *Dusp2, Egr1, Lpar3*, *Plk3,* and *Rasl11b* are also stimulators of monocyte differentiation and inflammatory responses [21-25].

Monocytes from aged rats also demonstrate up-regulated gene expression of the secretable cytotoxic factors GZMBL3, GZMF, and LYZL4. Granzymes are normally not regarded as monocyte-derived. However, several groups have shown that granzyme B is also produced by monocytes [26, 27]. Vascular research has been limited to this enzyme, which was found in the intima and media of advanced atherosclerotic lesions [28]. In addition, a recent work demonstrated that granzyme B increased vascular permeability by releasing vascular endothelial growth factor (VEFG) from the ECM [29]. In the setting of restenosis, it is possible that granzymes contribute to excessive VSMC cytotoxicity, which compromises the healing response of the wall and exacerbates inflammation [30]. Similarly, lysozyme M-positive monocytes are known to amplify oxidative stress in the vascular wall [31], suggesting that other lysozymes may also increase inflammation.

In contrast to the high number of adhesion molecules, chemotactic factors and inflammatory mediators overexpressed in monocytes from aged rats, up-regulated genes in cells isolated from young rats do not suggest a pro-inflammatory profile (Table 2 and Results). Instead, up-regulation of MHC class I receptor genes (*RT1*) and *S1p1r* indicate increased involvement in antigen presentation processes and trafficking to and from lymphoid organs [32]. Likewise, monocytes from young rats demonstrate up-regulated expression of the TLR3 and TLR4 intracellular adaptor TICAM-1, which likely relates to the increased response to bacterial LPS that is normally observed in young individuals [11].

Of note, a number of similarities in gene expression can be observed between monocytes from aged rats and pro-inflammatory CD16^+^ subpopulations in humans. These include up-regulation of granzymes, *Dusp2, Egr1, Gfra2*, and *Klrb1* [26, 33, 34]. Interestingly, higher numbers of these subpopulations are found in the elderly and in individuals with chronic inflammatory conditions [9, 12], where they have been associated with atherosclerosis and restenosis [12, 14, 15]. In addition, multiple pro-atherogenic factors are overexpressed in monocytes from aged animals, including *Cxcl1, Lpar3,* and the secretable lipases *Pla2g2f* and *Pnliprp2.* Macrophage-derived lipases are known to induce foam cell formation [35]. CXCL1 is also involved in atherosclerosis [7], and LPAR3 is required for monocyte recruitment to atherosclerotic lesions by oxidized LDL [36].

The overexpression of inflammatory mediators, cytotoxic factors, and adhesion molecules in monocytes from aged rats fits two different but complementary theories of aging: the hyperfunction theory [37] and inflammaging [38]. In fact, the former theory explains the latter and postulates that increased functions in both immune and non-immune cells lead to a loss of homeostasis, chronic inflammation and age-related diseases.

The limitations of this study include the possibilities that cellular contaminants were introduced as part of our monocyte isolation procedures, and that the identified mRNA expression profiles do not correspond to protein levels as a result of post-translational regulation. In addition, it is conceivable that perivascular delivery of high numbers of monocytes using a Matrigel system might over-promote their migration into the wall, compared to physiological conditions. Despite these limitations, our study demonstrates that monocytes from aged rats, but not young animals, induce restenosis after arterial injury. Furthermore, we show that their gene expression profile not only explains this observed inflammatory and restenotic character, but also predicts a highly pro-atherogenic potential.

## MATERIALS AND METHODS

### Isolation and purification of monocytes

Aged Fischer (>22-month-old, F344) rats were purchased from the National Institute of Aging (Bethesda, MD), while young (2-month-old) rats were obtained from Harlan Laboratories (Indianapolis, IN). Blood monocytes were isolated using density gradient followed by negative immunomagnetic separation [39]. Briefly, non-monocytic cells from Histopaque-1087 gradients were labeled with specific monoclonal antibodies (20 μg of Ox-8, Ox-19, Ox-33, Ox-52, anti-CD4, and anti-granulocytes from AbD Serotec) and discarded using anti-mouse magnetic beads. Monocytes purity was determined by FACS analysis using fluorescently labeled anti-CD4, -CD8, -CD11b, and -CD18 antibodies (AbD Serotec).

### Balloon injury and perivascular cell delivery

The Institutional Committee for Use and Care of Laboratory Animals at the University of Miami previously approved all animal procedures. Balloon injury in the right iliac artery was inflicted with a 2F Fogarty catheter (Baxter Corp., Irvine, CA, USA) adapted to a custom angiographic kit (Boston Scientific, Scimed) [40]. Approximately 2×10^6^ monocytes from either aged or young rats were re-suspended in 0.2 ml of BD Matrigel™ Matrix (BD Biosciences, San Jose, CA) and seeded around iliac arteries prior to balloon injury. Control animals received a similar volume of Matrigel alone. Arterial specimens were collected 21 days after injury and fixed in 4% formalin-PBS (Sigma-Aldrich). The areas of the neointima and media layers were measured in H&E stained slides using the ImagePro software (Media Cybernetics, Bethesda, MD).

### Immunohistochemistry

Paraffin sections were rehydrated and treated with 3% hydrogen peroxide to block endogenous peroxidase activity. Sections were boiled in citrate buffer to allow epitope retrieval. Non-specific binding sites were blocked with 0.5% blocking solution (DAKO, Carpinteria, CA) before adding primary antibodies against rat CD68 (AbD Serotec) and smooth muscle cell actin (DAKO) for 1 h at room temperature. Biotinylated secondary antibodies (DAKO Universal Link) were applied for 30 min, followed by a 15-min incubation with horseradish peroxidase-streptavidin solution (DAKO) at room temperature. Color was developed with a DAB chromogenic solution (DAKO). Nuclei were counterstained with Meyer's hematoxylin and mounted as described above.

Fresh isolated monocytes were cytospun onto microscope slides and fixed with cold methanol for 10 min at −20°C. Non-specific binding sites were blocked with 10% goat serum (Chemicon International, Temecula, CA) in PBS for 1 h at room temperature. Primary rabbit polyclonal antibodies against cadherin 13 and secreted frizzled-related protein 1 (Santa Cruz Biotechnology) were diluted in blocking solution and applied onto sections overnight at 4°C. After washing twice with PBS for 3 min, tissue sections were incubated with Alexa Fluor 488 (or 546) goat anti-rabbit (Invitrogen, Carlsbad, CA) for 90 min at room temperature. Sections were mounted in Vectashield w/DAPI (Vector Laboratories, Burlingame, CA) and examined with an Olympus BX 40 microscope (Olympus America Inc., Center Valley, PA).

### RNA microarray analysis

Total RNA from fresh isolated monocytes was extracted using TRI Reagent (Molecular Research Center, Cincinnati, OH), followed by an extra-cleaning step with the RNeasy kit (Qiagene, Valencia, CA). Gene expression profiling and data analyses were performed at Ocean Ridge Biosciences (Palm Beach Gardens, FL) using two-color Agilent Whole Rat Genome Arrays (Agilent Technologies). Differentially expressed genes were those with p-values and false discovery rates (FDR) under 0.05. Microarray results for selected genes were confirmed using TaqMan Gene Expression Assays (Applied Biosystems, Foster City, CA). Real-time PCR was performed on an ABI Prism 7500 Fast Real-Time PCR System and relative gene expression was determined using the ddCT method [41].

### Statistical analyses

Results were expressed as means ± SE. Two-group comparisons were conducted using two-tailed t-tests for independent samples with unequal variances. Statistics were calculated with Prism 5 (GraphPad Software, La Jolla, CA).
